# Implementation of collaborative care for depressive disorder treatment among accountable care organizations

**DOI:** 10.1097/MD.0000000000026539

**Published:** 2021-07-09

**Authors:** Helen Newton, Susan H. Busch, Mary Brunette, Donovan T. Maust, James O’Malley, Ellen R. Meara

**Affiliations:** a Department of Health Policy and Management, Yale School of Public Health, New Haven, CT; b Department of Psychiatry, Geisel School of Medicine at Dartmouth; c Bureau of Mental Health, NH Department of Health and Human Services, Concord, NH; d Department of Psychiatry, University of Michigan School of Medicine; e VA Ann Arbor Center for Clinical Management Research, Ann Arbor, MI; f The Dartmouth Institute for Health Policy and Clinical Practice; g Department of Biomedical Data Science, Geisel School of Medicine at Dartmouth, Lebanon, NH; h Harvard TH Chan School of Public Health; i National Bureau of Economic Research, Boston, MA.

**Keywords:** accountable care organizations, delivery system reform, mental health, primary care

## Abstract

Collaborative care – primary care models combining care management, consulting behavioral health clinicians, and registries to target mental health treatment – is a cost-effective depression treatment model, but little is known about uptake of collaborative care in a national setting. Alternative payment models such as accountable care organizations (ACOs), in which ACOs are responsible for quality and cost for defined patient populations, may encourage collaborative care use.

Determine prevalence of collaborative care implementation among ACOs and whether ACO structure or contract characteristics are associated with implementation.

Cross-sectional analysis of 2017–2018 National Survey of ACOs (NSACO). Overall, 55% of ACOs returned a survey (69% of Medicare, 36% of non-Medicare ACOs); 48% completed at least half of core survey questions. We used logistic regression to examine the association between implementation of core collaborative care components – care management, a consulting mental health clinician, and a patient registry to track mental health symptoms – and ACO characteristics.

Four hundred five National Survey of ACOs respondents answering questions on collaborative care implementation.

Only 17% of ACOs reported implementing all collaborative care components. Most reported using care managers (71%) and consulting mental health clinicians (58%), =just 26% reported using patient registries. After adjusting for multiple ACO characteristics, ACOs responsible for mental health care quality measures were 15 percentage points (95% CI 5–23) more likely to implement collaborative care.

Most ACOs are not utilizing behavioral health collaborative care. Including mental health care quality measures in payment contracts may facilitate implementation of this cost-effective model. Improving provider capacity to track and target depression treatment with patient registries is warranted as payment contracts focus on treatment outcomes.

## 1 Introduction

Untreated depression can be physically and financially disabling; patients with depressive disorders are more likely to have chronic medical conditions like diabetes and cardiovascular disease, with medical expenditures nearly double that of individuals without depression.^[1–6]^ Yet, less than 30% of people screening positive for depression actually receive treatment.^[6]^ Care that integrates mental health treatment into primary care settings – behavioral health collaborative care – improves access to high quality depression treatment, as demonstrated by the Improving Mood-Promoting Access to Collaborative Treatment model.^[7,8]^ Such collaborative care models typically include 3 core structural components:

1) care management,
2) a consulting mental health clinician (often a psychiatrist) to support the primary care provider,
3) and a patient registry to track mental health symptoms and facilitate population mental health management.^[7]^

These models are effective for a variety of behavioral health conditions and they lead to improved outcomes and quality of life, yet they have been challenging to implement outside of clinical trial settings due to reliance on traditionally non-billable services, such as care management and staff that are often in short supply.^[9–11]^

Providers participating in alternative payment models– like accountable care organizations (ACOs) – are often early adopters of delivery system innovations and may be more likely to implement behavioral health collaborative care.^[12]^ ACOs are groups of doctors, hospitals, or other health care providers who are held responsible for the total cost and quality of health care services on an assigned patient population through an ACO contract.^[13]^ ACO contracts represent an important shift away from the traditional fee-for-service health care delivery model by tying financial performance to health care quality, with performance measures aimed at limiting utilization in high-resource settings and facilitating care coordination. Some ACO contract features prioritize populations with mental illness. For example, both Medicare and Massachusetts’ Alternative Quality Contract include quality measures related to depression management in their ACO contracts.^[14]^

Aside from several notable case studies, national surveys examining early ACO efforts to integrate mental health into primary care settings showed that integration efforts were limited, though many ACOs reported additional focus on patients with mental illness.^[15–18]^ Analyses of Medicare beneficiaries attributed to early ACO participants saw little change in mental health service utilization and only moderate improvement in antidepressant adherence.^[19,20]^ These effects were largely concentrated among ACOs participating in models with downside financial risk, like Medicare's Advanced Payment or Pioneer ACO Models, and ACOs partnering with safety-net organizations.^[17–20]^ These early findings demonstrated that ACO contract features may be associated with integrated care delivery even though few organizations reported integrating mental health and primary care. Still unknown, however, was the extent to which ACOs were using evidence-based models to integrate mental health and primary care – models like collaborative care – and whether and how other ACO characteristics were associated with the use of evidence-based care delivery. And as ACO models mature, the extent of collaborative care could change.

To update and expand our understanding of how ACOs integrate mental health and primary care, our study uses the 2017–2018 National Survey of ACOs (NSACO), the largest survey of ACOs to-date, to examine how and whether ACOs implemented collaborative care to integrate mental health and primary care. We focus on ACOs because these organizations are often innovators in care delivery and are the providers most likely to implement cost-effective models like collaborative care. If ACOs are not using collaborative care, it is likely that other providers and organizations also find implementation difficult. By understanding the correlates of collaborative care use, our evidence can offer clues as to what could facilitate future integration of mental health and primary care.

## 2 Methods

### 2.1 Study design

We conducted a cross-sectional analysis using responses to the 2017–2018 NSACO to determine the extent to which ACOs have implemented collaborative care models and identify ACO characteristics associated with implementation.

### 2.2 National survey of ACOs

We fielded the 2017–2018 NSACO to potential respondents from July 2017 to March 2018. The survey instrument included questions about ACO contract payers, organizational characteristics, and treatment delivery capabilities specific to mental health care delivery and ACO contract features. We fielded a web and paper version of the instrument to improve response rate; most (80%) of respondents completed the survey online versus paper. The intended respondents were personnel most knowledgeable about ACO contract administration, such as a chief executive or medical officer, population health administrator. The Dartmouth College Committee for the Protection of Human Subjects approved the survey protocol and all communication with potential survey respondents, including informed consent.

### 2.3 Setting and participants

**Figure 1:**
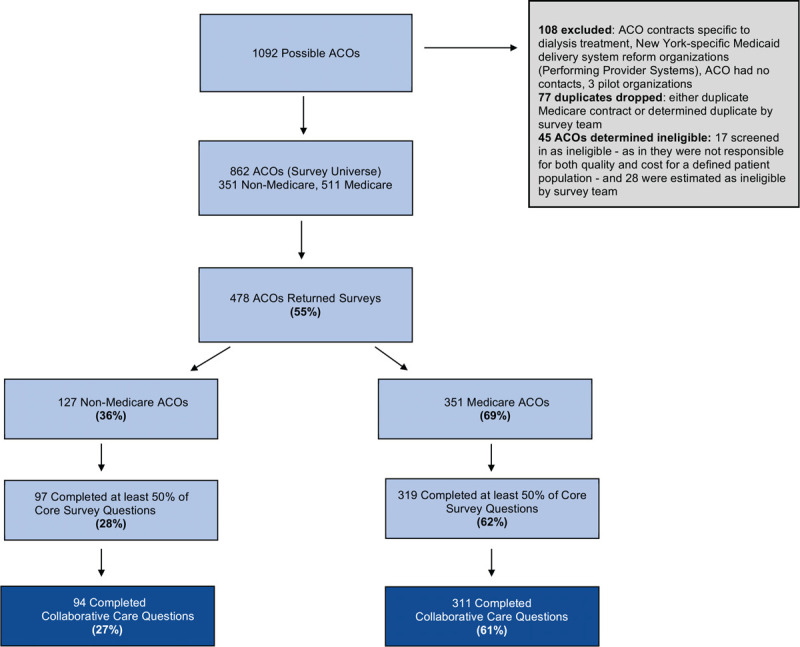
Participants in 2017–2018 National Survey of ACOs. *Notes*: This figure shows the sampling frame of the 2017–2018 National Survey of ACOs. Of 862 eligible ACOs, 478 (55%) returned a survey and 405 (47%) completed collaborative care questions. ACO = accountable care organization.

We defined an ACO as a provider organization participating in contracts that held them responsible for the total cost of care and quality of care for a designated patient population, consistent with the definition used in previous waves of the NSACO and by the Centers for Medicare and Medicaid Services.^[13,21]^ We considered all organizations participating in 2017 and 2018 ACO contracts potential survey respondents (Fig. 1). We excluded dialysis treatment organizations, New York Performing Provider Systems, organizations without contact information, organizations not meeting our definition of an ACO (ineligible) and organizations surveyed during survey instrument development (our pilot group). Of an estimated 862 eligible provider organizations participating in ACO contracts, 55% returned a survey and 48% answered at least half of the core questions. Response rates were higher among organizations with a Medicare contract: 69% of organizations with a Medicare contract returned a survey, while 36% of ACOs without Medicare contracts returned surveys (non-response analysis available, p. 17 in Supplement). Our analysis used responses from the 405 ACOs who responded to questions asking how providers in their ACO integrated mental health and primary care for patients with depressive disorders.

### 2.4 Main outcome measure

Our outcome of interest was implementation of collaborative care, which we operationalized based on components endorsed in response to this survey question: “Do any providers in your ACO use [component x] to integrate primary care and treatment for depression and/or anxiety?” Options the respondents could endorse included:

1) a care manager for mental health or non-medical needs;
2) a consulting mental health clinician; and
3) a patient registry to track mental health symptoms.

“Full” implementation of collaborative care required endorsing all 3 components (full text p. 2–3 in Supplement).

Due to the inadvertent substitution of “physical health” for “mental health” in the care management question in our web-based survey, this question differed in the paper versus web-based survey. We addressed this discrepancy in several ways to confirm that our results were not sensitive to the question wording. We first excluded all web responses to the incorrectly worded care management survey question (79% of total responses). We then created a composite care management variable that combined responses to survey questions asking about all care manager roles. Finally, we conducted extensive sensitivity analyses to test whether results were sensitive to the specification of our care management outcome (p. 15–16 in Supplement). Because nearly all respondents report using a care manager, ACOs were distinguished much more by their use of consulting mental health clinicians or use of a registry to track patients with depression; thus, our model results changed little with the exclusion of the care management variable.

### 2.5 Covariates

We included ACO contract and organizational characteristics measured in the 2017–2018 NSACO as covariates in our analysis. Contract characteristics included the payer(s) with whom respondents had contracts (Medicare, Medicaid, or commercial payers), whether the respondent shared financial risk in any contract (e.g., took on downside risk), and whether the respondent had previous experience with risk-based contracting. We also measured contract features specific to mental health services in respondents’ non-Medicare ACO contracts, because unlike Medicare, commercial and Medicaid ACO contracts are not required to include mental health services. These included whether the contract included mental health services in the total cost of care calculation (the financial benchmark), whether the contract's performance measure set included any mental health care quality measures and whether the contract financially “carved-out” mental health services, by contracting out care to a managed behavioral health care organization. We included 2 organizational characteristics to account for potential differences in organizational priority and capacity to integrate mental health treatment into primary care: self-reported leadership (physician-led organizations versus non-physician-led) and organizational size. ^[21,22]^ We operationalized size as total number of primary care and specialty physician full time equivalents (FTEs) participating in the ACO, by quartile. All other covariates were indicator variables (full question text in Supplement, p. 3–8).

### 2.6 Statistical analysis

We measured the proportion of ACOs who reported using each of 3 collaborative care components – care manager, consulting mental health clinician, or patient registry – and then measured the proportion of ACOs who reported using no collaborative care components, any 1 or 2 components (partial implementation), or all 3 components (full implementation). We compared the distribution of ACO characteristics among ACOs who reported full implementation of collaborative care versus those with partial or no implementation. We examined the association between ACO characteristics on implementation of any collaborative care component using logistic regression. Our outcome of interest was the likelihood of an ACO implementing any collaborative care component (1 = use of a core collaborative care component [e.g., a care manager, a consulting mental health clinician, or a patient registry], 0 = reported not using a core collaborative care component). Because we measured ACO's use of 3 collaborative care components, we treated each component as a multivariate outcome. This approach made full use of the information included in the data and estimated the correlation between collaborative care components while automatically accounting for the multiple measurements made on each ACO. We created an additional predictor variable to indicate the type of collaborative care component (a 3-level categorical variable; 1 = outcome variable refers to use of a care manager, 2 = consulting mental health clinician, 3 = patient registry). We included ACO characteristics as covariates and specified interaction terms between the component type and each ACO characteristic to identify how and whether the type of component affected associations between ACO characteristics and likelihood of collaborative care implementation. We fit our logistic regression model using generalized estimating equations with an unstructured correlation matrix to allow different correlations between the adoption of each pair of components and robust standard errors.^[23]^ We estimated the “population averaged effect,” or the log-odds of implementing any collaborative care component, accounting for the correlation between using multiple components within each ACO (model output in Supplement, p. 11–12).^[24]^ We considered *P* ≤ .05 statistically significant and all statistical analysis was conducted using Stata/IC 15.1.

### 2.7 Missing data

Fifty-two respondents (13%) skipped at least 1 question when answering the survey. We accounted for missing covariate data using multiple imputation. We used all covariates included in our analytic model in our imputation model and specified 10 datasets across which to pool final estimates. Our results in the text show estimates using the multiply imputed data, though we conducted sensitivity analyses to compare model results when restricting results to complete case data (p. 14 in Supplement).

## 3 Results

### 3.1 Characteristics of ACOs 2017–2018

In the 2017–2018 NSACO, 405 ACOs answered how providers in their ACO integrated mental health and primary care services for patients with depressive disorders in the 2017–2018 NSACO (Fig. 1). Most ACOs had contracts with Medicare (83%) and commercial payers (73%) while just a quarter had an ACO arrangement with Medicaid (24%; Table 1). Most ACOs had prior experience with risk-based contracts (64%), but just a third (37%) reported sharing financial risk in any contract. Less than half of ACOs included mental health services in either the financial benchmark or performance measure set for non-Medicare contracts (43% and 34%, respectively). Just 20% reported financially carving out mental health services from their non-Medicare contracts to managed behavioral health organizations. Just over a third (38%) of ACOs were physician-led. Few ACOs reported including safety-net organizations – such as specialty behavioral health providers, Federally Qualified Health Centers, or public hospitals – in their ACO networks (Table 1).

**Table 1:** Characteristics of ACOs fully implementing collaborative care compared to those either partially or not implementing.

	Whole sample	Collaborative care implementation			
		None (0 strategies)	Partial (1–2 strategies)	Full (3 strategies)	
	(405 ACOs, 100%)	(65 ACOs, 16%)	(271 ACOs, 67%)	(69 ACOs, 17%)	*P*
Contract characteristics					
Payer, N (%)					
(Most ACOs have contracts with 2 or more payers)					
Has a Medicare contract	338 (83%)	56 (86%)	226 (84%)	56 (80%)	.62
Has a commercial contract	295 (73%)	40 (62%)	201 (74%)	54 (77%)	.08
Has a Medicaid contract	96 (24%)	6 (9%)	68 (25%)	22 (31%)	.01
Financial characteristics, N (%)					
Shares financial risk in any contract^∗^	149 (37%)	16 (25%)	98 (36%)	35 (50%)	.01
Previous experience in risk-based contracts	257 (64%)	35 (54%)	175 (66%)	47 (69%)	.15
Mental health contract characteristics, N (%)					
(included in non-Medicare contracts)					
Includes mental health services in total cost of care calculation	163 (43%)	17 (29%)	111 (44%)	35 (52%)	.02
Includes mental health in quality performance measures	134 (34%)	7 (11%)	99 (37%)	28 (41%)	<.01
Carves out mental health services from contract	84 (21%)	7 (11%)	57 (21%)	20 (29%)	.04
Organizational characteristics					
Leadership, N (%)					
Physician-led ACO^∗^	143 (38%)	36 (56%)	83 (33%)	24 (39%)	<.01
Partnerships, N (%)					
Includes specialty behavioral health provider in ACO network^∗^	54 (13%)	3 (5%)	35 (13%)	16 (23%)	.01
Includes federally qualified health center (FQHC) in ACO network	104 (26%)	14 (22%)	69 (26%)	21 (31%)	.50
Includes academic medical center in ACO network	72 (18%)	3 (5%)	52 (21%)	14 (21%)	.02
Includes public hospital in ACO network	45 (11%)	8 (13%)	26 (10%)	11 (16%)	.33
Size mean (95% CI)					
Number of clinicians in ACO network	797 (679–915)	408 (218–599)	855 (710–1000)	935 (595–1275)	.01

∗ *Notes*: This figure shows data from the 405 ACOs who reported their use of collaborative care strategies in the 2017–2018 NSACO. We imputed missing covariate data using multiple imputation. We considered all ACOs participating in Medicare Shared Savings Program Tracks 1+, 2, or 3 and those participating in Medicare's Next Gen ACOs as sharing financial risk, in addition to those who reported taking on downside risk in their commercial or Medicaid contracts. We considered organizations physician-led if they reported physician leadership and did not include a hospital in their network. Specialty behavioral health provider refers to community mental health centers and addiction treatment centers. We used an *F* tests for significance testing between levels of collaborative care implementation. ACO = accountable care organization; FQHC = federally qualified health center; NSACO = National Survey of ACOs. *Source*: 2017–2018 NSACO.

### 3.2 ACO implementation of collaborative care 2017–2018

**Figure 2:**
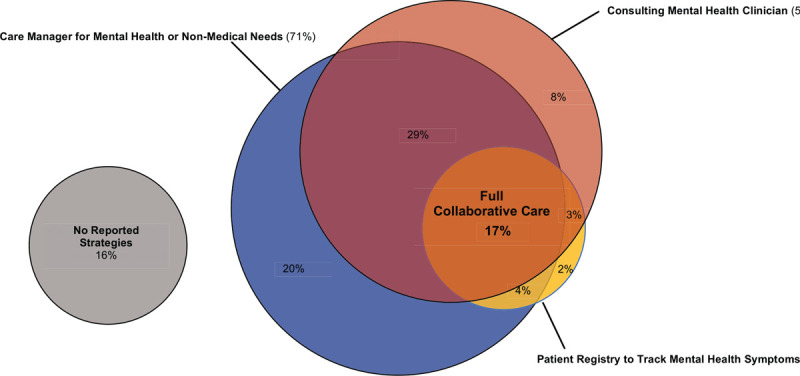
Implementation of collaborative care strategies in accountable care organizations 2017–2018. *Notes*: This figure shows the distribution of collaborative care implementation for the 405 ACOs who reported their use of collaborative care to integrate mental health and primary care services in the 2017–2018. We imputed missing outcome data for 3 ACOs using multiple imputation. ACO = accountable care organization; NSACO = National Survey of ACOs. *Source*: 2017–2018 NSACO.

Just 17% of ACOs reported using all 3 components of collaborative care, or full collaborative care implementation (Fig. 2). Most ACOs (84%), however, reported using at least 1 component. Most ACOs reported using a care manager (71%) or a mental health clinician to consult the primary care provider (58%), but only a quarter of ACOs (26%) reported using a patient registry to track mental health symptoms for patients with depression or anxiety (Fig. 2).

### 3.3 Characteristics of ACOs implementing collaborative care

ACOs fully implementing collaborative care were more likely to have an ACO arrangement with Medicaid (31%) than ACOs who used just 1 to 2 components (25%) or no components (9%) (*P* = .01). These organizations were also more likely to include mental health services in the total cost of care calculation (52% vs 44% of those using 1–2 components and 29% using no components, *P* = .02) and mental health care quality measures (41% vs 37% and 11%, *P* < .01) in non-Medicare contracts. These organizations were also more likely share financial risk in any contract (50% vs 36% and 25%, *P* = .01; Table 1).

ACOs fully implementing collaborative care were more likely to include specialty behavioral health care providers in their ACO networks compared to ACOs using few or no collaborative care components (23% vs 13% and 5% respectively, *P* = .01). A greater proportion of the ACOs fully implementing collaborative care reported Federally Qualified Health Centers and public hospitals in their ACO networks compared to other ACOs, but these differences were not statistically significant. ACOs implementing any collaborative care components were less likely to be physician-led than those using no collaborative care components (39% and 33% vs 56%, *P* < .01). ACOs fully implementing collaborative care were larger on average than ACOs partially or not implementing collaborative care, employing on average 935 clinical FTEs versus 855 and 408, respectively (*P* = .01; Table 1).

### 3.4 Adjusted association of ACO characteristics with collaborative care implementation

**Figure 3:**
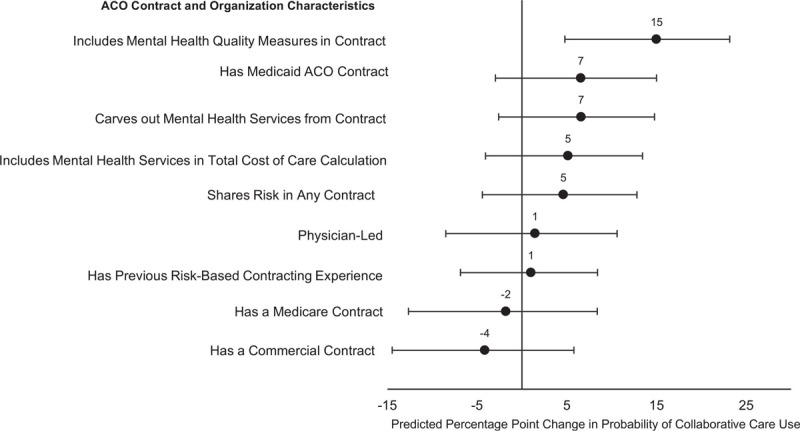
Association between ACO contract and organizational characteristics and predicted probability of collaborative care implementation. *Notes*: This figure shows the association between ACO contract and organization characteristics and predicted use of collaborative care strategies for the 405 ACOs who reported their use of collaborative care in the 2017–2018 NSACO. We considered use of the 3 collaborative care strategies as a repeated outcome clustered within each ACO and used a logistic regression fit with generalized estimating equations to account for this correlation. All coefficients listed in this figure were included in the model, as well as variables for ACO size, the type of collaborative care strategy, the type of survey instrument used (web or paper), and 2 interaction terms (interactions between the type of strategy and use of mental health care quality measures in non-Medicare ACO contracts and the type of strategy and ACO size). We imputed missing covariate information using multiple imputation. Covariates that indicate specific mental health care contract characteristics (inclusion of mental health services in the total cost of care, in quality measures, or carving out mental health services) are only applicable to organizations with either a Medicaid and/or a commercial ACO contract (73% of respondents). ACO = accountable care organization; NSACO = National Survey of ACOs. *Source*: 2017–2018 NSACO.

**Figure 4:**
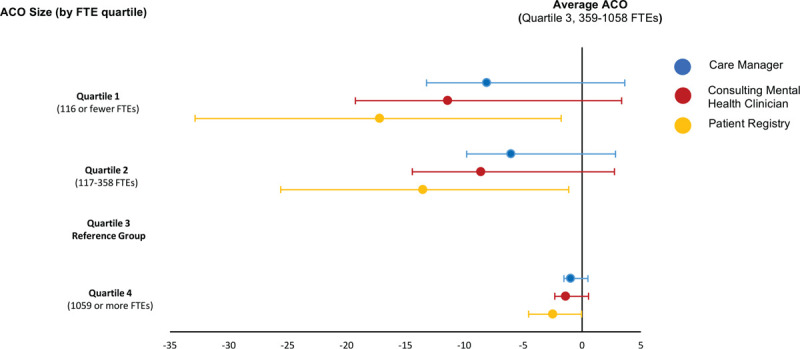
Association between organizational size and predicted probability of collaborative care implementation by strategy type. *Notes*: Using the same regression model shown in Figure 3, this figure shows effect of an ACO's size (number of physician full time equivalents, or FTEs) and the change in likelihood of using collaborative care strategies compared to the average ACO for the 405 ACOs who reported their use of collaborative care in the 2017–2018 NSACO. This figure demonstrates that smaller organizations are less likely to use collaborative care strategies compared to larger organizations, particularly patient registries. ACO = accountable care organization; FTEs = full time equivalents; NSACO = National Survey of ACOs. *Source*: 2017–2018 NSACO.

After adjusting for multiple ACO characteristics, organizations including mental health care quality measures in a non-Medicare contract were 15 percentage points more likely to implement collaborative care (95% confidence interval 5–23; Fig. 3). Other characteristics associated with full collaborative care implementation in unadjusted analyses, such as payer-type, sharing financial risk, or participating in a physician-led ACO, had no significant relationship with collaborative care implementation in the adjusted model. Smaller organizations (those in the bottom 2 size quartiles, or those with fewer than 359 physician FTEs) were less likely to report using collaborative care components than average-sized organizations (third size quartile, the reference group), though there was no difference between the largest organizations and those of average size (Fig. 4).

## 4 Discussion

In this study, we present 2017–2018 data from a national survey of ACOs to show how and whether these organizations implement behavioral health collaborative care. These data are the most recent available and we survey mature organizations that have had multiple years to develop collaborative care models. Yet, we found that just 17% of ACOs report using all 3 components of behavioral health collaborative care, though most do report using at least 1 component. The 2017–2018 survey builds upon prior literature because of its large sample, 405 ACOs, because it took place when organizations had more experience with ACO contracts, and because it is the only survey of a large national sample of ACOs to ask detailed questions about ACO structure, organization, and approaches to integrating primary and mental health care.

Despite 15 years of robust evidence supporting use of collaborative care, its team-based services did not fit neatly into a traditional fee-for-service payment model until changes in payment rules encouraged such care in 2017.^[25–28]^ ACO contracts could help to overcome these financial barriers by holding participating providers accountable for the total cost and quality of care and encouraging adoption of cost-effective care delivery strategies to manage chronic disease. While surveys of early ACO participants found that few ACOs (14%) had integrated behavioral health into their primary care settings, we would have expected that respondents to our survey, fielded 5 years after these first surveys, to be more likely than these early participants to report implementation of collaborative care. Yet, we found that fewer than 1 in 5 ACOs were fully implementing collaborative care, and few contract characteristics were associated with implementation.

We found no association between the level of financial risk included in the ACO contract and collaborative care implementation. Earlier surveys of ACOs, when Medicare first launched its ACO models, found that ACOs participating in contracts facing downside financial risk, those that shared losses when spending was above benchmarks set for their assigned population, were more likely to report taking on initiatives to integrate behavioral health and primary care.^[17]^ These same ACOs were also more likely to see improvements in antidepressant adherence and increases in in-network mental health treatment utilization.^[19,20]^ We similarly expected 2017–2018 ACOs facing downside financial risk in their ACO contracts to be more likely to implement collaborative care strategies than other ACOs. Instead, we found that facing greater financial risk was unrelated to collaborative care use in adjusted models. Our findings imply that the recent changes to Medicare's Shared Savings Program, which encourage quicker transition to risk-sharing contracts, will not necessarily enable collaborative care adoption.

Unlike prior surveys, we specifically asked whether ACOs used patient registries to track mental health symptoms and target treatment in addition to other collaborative care strategies. This difference in measurement may account for our finding that collaborative care implementation remains limited. Patient registries are essential to collaborative care through facilitating treatment for a panel of patients, encouraging communication between clinicians, and tracking symptom progress until treatment goals are reached. We found that just 26% of ACOs reported using a patient registry to track and target mental health treatment, less than half the proportion reporting use of care managers or consulting mental health clinicians. This finding corroborates concerns that ACOs have difficulty reporting on the depression remission measure introduced in 2016 for Medicare ACO contracts and underscores the real challenges in clinical workflow and staffing that registry use brings.^[25]^ Counts and colleagues reported that 32% of Medicare ACOs did not report their depression remission measure in 2017, even though it is pay-for-reporting only.^[29]^ Those that did report reported much lower rates of remission than found in clinical trials (median 8%).^[29,30]^ Counts and colleagues suggested that poor performance could be explained by poor patient follow-up.^[29]^ Our findings suggest that ACOs may also have difficulty tracking patients’ mental health symptoms required to report remission.

Our study offers insights on how alternative payment models like ACOs might affect use of evidence-based practice to integrate mental health and primary care in the future. Health systems and payers will likely continue to focus on behavioral health and primary care integration, in part because the savings attributed to integrating care delivery is high – over $31 billion.^[31]^ At the same time, providers increasingly face mandated participation in payment contracts that tie financial payment to quality of patient care. We found that ACOs who reported mental health care quality measures in their non-Medicare contracts were 15 percentage points more likely to implement collaborative care, suggesting that mental health care quality measures may be an integral component of new payment contracts seeking to maximize mental health and primary care integration.

Our findings imply too that population health management of behavioral health conditions requires more than just innovative contract design. The lag in adoption of patient registries, even with new collaborative care billing codes and quality measures encouraging outcome tracking, suggests that ACOs may benefit from targeted quality improvement efforts. For example, Medicare's advanced primary care payment model, comprehensive primary care plus, provides participating practices with technical assistance for quality improvement in areas like depression treatment, in addition to flexible financial resources.^[32]^ Adding additional depression care quality measures alone, without improving capacity to track outcomes and treat to target, may just penalize organizations rather than lead to meaningful changes in health care delivery and outcomes for adults with depressive disorders.

### 4.1 Strengths and limitations

This is the first study that we know to explicitly examine behavioral health collaborative care implementation and associated mental health specific contract characteristics in a national sample of ACOs. Importantly, this study uses national data from the largest survey of ACOs to-date and includes responses from ACOs with contracts from all payers. However, this study has several important limitations. Our estimated number of ACOs implementing collaborative care may be inflated due to potential measurement error and social desirability bias inherent in self-reported data. The error in our care management survey question may have led to an underestimate of the ACOs reporting use of care managers because all web responses to this survey question (79%) were excluded. Finally, the number of ACO contracts has increased since we fielded the survey (July 2017–February 2018) with potentially different contract features.

## 5 Conclusion

We found that 17% of ACOs reported full implementation of behavioral health collaborative care in their primary care settings. Given the apparent challenge of using patient registries to track patient-reported mental health symptoms, payers interested in incentivizing integrated primary care–mental health treatment should address these barriers to collaborative care implementation, including strengthening incentives and creating targeted quality improvement efforts that prioritize investment in registries, outcome tracking, and treating to target.

## Author contributions

**Conceptualization:** Helen Newton, Susan H Busch, Mary Brunette, Donovan Maust, James O’Malley, Ellen R Meara.

**Data curation:** Helen Newton.

**Formal analysis:** Helen Newton.

**Funding acquisition:** Ellen R Meara.

**Methodology:** James O’Malley, Ellen R Meara.

**Supervision:** Susan H Busch, Mary Brunette, Donovan Maust, James O’Malley, Ellen R Meara.

**Visualization:** Helen Newton.

**Writing – original draft:** Helen Newton.

**Writing – review & editing:** Helen Newton, Susan H Busch, Mary Brunette, Donovan Maust, James O’Malley, Ellen R Meara.

## Supplementary Material

Supplemental Digital Content

## References

[1] de Groot M Anderson R Freedland KE Clouse RE Lustman PJ. Association of depression and diabetes complications: a meta-analysis. Psychosom Med 2001;63:619–30. 11485116 10.1097/00006842-200107000-00015

[2] Sussman M O'sullivan AK Shah A Olfson M Menzin J. Economic burden of treatment-resistant depression on the U.S. health care system. J Manag Care Spec Pharm 2019;25:823–35. DOI 10.18553/jmcp.2019.25.7.823. 31232205 10.18553/jmcp.2019.25.7.823 PMC10398213

[3] Thorpe K Jain S Joski P. Prevalence and spending associated with patients who have a behavioral health disorder and other conditions. Health Aff (Project Hope) 2017;36:124–32. DOI 10.1377/hlthaff.2016.0875. 10.1377/hlthaff.2016.0875 28069855

[4] Walker ER Druss BG. Cumulative burden of comorbid mental disorders, substance use disorders, chronic medical conditions, and poverty on health among adults in the U.S.A. Psychol Health Med 2017;22:727–35. DOI 10.1080/13548506.2016.1227855. 27593083 10.1080/13548506.2016.1227855 PMC5564203

[5] Egede LE Bishu KG Walker RJ. Impact of diagnosed depression on healthcare costs in adults with and without diabetes: United States, 2004–2011. J Affect Disord 2016;195:119–26. DOI 10.1016/j.jad.2016.02.011. 26890289 10.1016/j.jad.2016.02.011 PMC4779740

[6] Olfson M Blanco C Marcus SC. Treatment of adult depression in the United States. JAMA Intern Med 2016;176:1482–91. DOI 10.1001/jamainternmed.2016.5057. 27571438 10.1001/jamainternmed.2016.5057

[7] Unützer J Katon W Callahan CM. Collaborative care management of late-life depression in the primary care setting: a randomized controlled trial. JAMA 2002;288:2836–45. 12472325 10.1001/jama.288.22.2836

[8] Katon WJ Lin EHB Von Korff M. Collaborative care for patients with depression and chronic illnesses. N Engl J Med 2010;363:2611–20. DOI 10.1056/NEJMoa1003955. 21190455 10.1056/NEJMoa1003955 PMC3312811

[9] Woltmann E Grogan-Kaylor A Perron B Georges H Kilbourne AM Bauer MS. Comparative effectiveness of collaborative chronic care models for mental health conditions across primary, specialty, and behavioral health care settings: systematic review and meta-analysis. Am J Psychiatry 2012;169:790–804. DOI 10.1176/appi.ajp.2012.11111616. 22772364 10.1176/appi.ajp.2012.11111616

[10] Carlo AD Unützer J Ratzliff ADH Cerimele JM. Financing for collaborative care – a narrative review. Curr Treat Options Psychiatry 2018;5:334–44. DOI 10.1007/s40501-018-0150-4. 30083495 10.1007/s40501-018-0150-4 PMC6075691

[11] Bagayogo IP Turcios-Wiswe K Taku K Peccoralo L Katz CL. Providing mental health services in the primary care setting: the experiences and perceptions of general practitioners at a New York City Clinic. Psychiatr Q 2018;89:897–908. DOI 10.1007/s11126-018-9587-2. 29968148 10.1007/s11126-018-9587-2

[12] Bao Y Casalino LP Pincus HA. Behavioral health and health care reform models: patient-centered medical home, health home, and accountable care organization. J Behav Health Serv Res 2013;40:121–32. DOI 10.1007/s11414-012-9306-y. 23188486 10.1007/s11414-012-9306-y PMC3568195

[13] Centers for Medicare & Medicaid Services. Accountable Care Organizations (ACOs). CMS.gov. Published October 2, 2019. Accessed January 21, 2020. https://www.cms.gov/Medicare/Medicare-Fee-for-Service-Payment/ACO.

[14] Barry CL Stuart EA Donohue JM. The early impact of the “Alternative Quality Contract” on mental health service use and spending in Massachusetts. Health Aff (Millwood) 2015;34:2077–85. DOI 10.1377/hlthaff.2015.0685. 26643628 10.1377/hlthaff.2015.0685 PMC4950854

[15] Reiss-Brennan B Brunisholz KD Dredge C. Association of integrated team-based care with health care quality, utilization, and cost. JAMA 2016;316:826–1826. DOI 10.1001/jama.2016.11232. 27552616 10.1001/jama.2016.11232

[16] Clarke RMA Jeffrey J Grossman M Strouse T Gitlin M Skootsky SA. Delivering on accountable care: lessons from a behavioral health program to improve access and outcomes. Health Aff (Millwood) 2016;35:1487–93. DOI 10.1377/hlthaff.2015.1263. 27503975 10.1377/hlthaff.2015.1263

[17] Fullerton CA Henke RM Crable E Hohlbauch A Cummings N. The impact of Medicare ACOs on improving integration and coordination of physical and behavioral health care. Health Aff (Project Hope) 2016;35:1257–65. DOI 10.1377/hlthaff.2016.0019. 10.1377/hlthaff.2016.0019 27385242

[18] Lewis VA Colla CH Tierney K Van Citters AD Fisher ES Meara E. Few ACOs pursue innovative models that integrate care for mental illness and substance abuse with primary care. Health Aff (Project Hope) 2014;33:1808–16. DOI 10.1377/hlthaff.2014.0353. 10.1377/hlthaff.2014.0353 25288427

[19] Busch AB Huskamp HA McWilliams JM. Early efforts by Medicare accountable care organizations have limited effect on mental illness care and management. Health Aff (Project Hope) 2016;35:1247–56. DOI 10.1377/hlthaff.2015.1669. 10.1377/hlthaff.2015.1669 27385241

[20] Busch AB Huskamp HA Kreider AR McWilliams JM. Medicare accountable care organizations and antidepressant use by patients with depression. Psychiatr Serv 2017;68:1193–6. DOI 10.1176/appi.ps.201600538. 28712357 10.1176/appi.ps.201600538 PMC5665698

[21] Colla CH Lewis VA Shortell SM Fisher ES. First national survey of ACOs finds that physicians are playing strong leadership and ownership roles. Health Aff (Millwood) 2014;33:964–71. DOI 10.1377/hlthaff.2013.1463. 24889945 10.1377/hlthaff.2013.1463

[22] McWilliams JM Hatfield LA Landon BE Hamed P Chernew NE. Medicare spending after 3 years of the Medicare shared savings program. N Engl J Med 2018;379:1139–49. DOI 10.1056/NEJMsa1803388. 30183495 10.1056/NEJMsa1803388 PMC6269647

[23] Zeger SL Liang KY. Longitudinal data analysis for discrete and continuous outcomes. Biometrics 1986;42:121–30. 3719049

[24] Norton EC Dowd BE Maciejewski ML. Marginal effects-quantifying the effect of changes in risk factors in logistic regression models. JAMA 2019;321:1304–5. DOI 10.1001/jama.2019.1954. 30848814 10.1001/jama.2019.1954

[25] Davis M Balasubramanian BA Waller E Miller BF Green LA Cohen DJ. Integrating behavioral and physical health care in the real world: early lessons from advancing care together. J Am Board Fam Med 2013;26:588–602. DOI 10.3122/jabfm.2013.05.130028. 24004711 10.3122/jabfm.2013.05.130028

[26] Bishop TF Ramsay PP Casalino LP Bao Y Pincus HA Shortell SM. Care management processes used less often for depression than for other chronic conditions in US primary care practices. Health Aff 2016;35:394–400. DOI 10.1377/hlthaff.2015.1068. 10.1377/hlthaff.2015.1068 26953291

[27] Katon W Unützer J Wells K Jones L. Collaborative depression care: history, evolution and ways to enhance dissemination and sustainability. Gen Hosp Psychiatry 2010;32:456–64. DOI 10.1016/j.genhosppsych.2010.04.001. 20851265 10.1016/j.genhosppsych.2010.04.001 PMC3810032

[28] Press MJ Howe R Schoenbaum M. Medicare payment for behavioral health integration. N Engl J Med 2017;376:405–7. DOI 10.1056/NEJMp1614134. 27973984 10.1056/NEJMp1614134

[29] Counts NZ Wrenn G Muhlestein D. Accountable care organizations’ performance in depression: lessons for value-based payment and behavioral health. J Gen Intern Med 2019;34:2898–900. DOI 10.1007/s11606-019-05047-x. Published online May 15. 31093839 10.1007/s11606-019-05047-x PMC6854154

[30] Khan A Faucett J Lichtenberg P. A systematic review of comparative efficacy of treatments and controls for depression. PLoS One 2012;7:e41778 DOI 10.1371/journal.pone.0041778. 22860015 10.1371/journal.pone.0041778 PMC3408478

[31] Shrank WH Rogstad TL Parekh N. Waste in the US health care system: estimated costs and potential for savings. JAMA 2019;322:1501–9. DOI 10.1001/jama.2019.13978. 31589283 10.1001/jama.2019.13978

[32] Comprehensive Primary Care Plus (CPC+): A Model for Primary Care in America. Centers for Medicare & Medicaid Services; 2017;2. https://innovation.cms.gov/initiatives/comprehensive-primary-care-plus.

