# Supplementary material for: Implementation of collaborative care for depressive disorder treatment among accountable care organizations
Source: Medicine (Baltimore). 2021 Jul 9;100(27):e26539. doi: 10.1097/MD.0000000000026539 (PMC8270614; doi:10.1097/MD.0000000000026539)
Supplement: Supplemental Digital Content [file medi-100-e26539-s001.docx]

**Supplemental Content**

# **Table of Contents**

### **Section 1:** 2017-2018 NSACO Survey Question Test………………..….….2

### **Section 2:** Statistical Technical Appendix…………………………….…….9

### **Section 3:** Missing Data Procedures………………………………… ....…..14

1. **Section 4:** Care Management Sensitivity Analysis…………………………..15
2. **Section 5:** Non-Response Analysis…………………………………………..17

### **Section 6:** Survey Mode Sensitivity Analysis………………..……..……….18

**Section 1: 2017-2018 National Survey of ACOs Question Text**

All variables included in study analyses were derived from responses to the 2017-2018 National Survey of Accountable Care Organizations. Specific question text and information on how variables were operationalized from question responses is described in table below:

**Outcome Variables: Collaborative Care**

The primary outcome in our analysis was reported use of collaborative care to integrate mental health and primary care services for patients with depression or anxiety. We derived this outcome using responses to four questions designed to capture the primary strategies included in the collaborative care, or IMPACT model (as defined by University of Washington AIMs center and assured by clinical collaborators on this project):

1) A care manager to manage mental health or non-medical needs ( a response of 1, or yes, to either question q192a or q192b):

q192a Do any providers in your ACO use a care manager to primarily address mental health treatment coordination to integrate primary care and treatment for **depression and/or anxiety**?

1 Yes

0 No

q192b Do any providers in your ACO use a care manager to address non-medical needs (e.g., job support, housing) to integrate primary care and treatment for **depression and/or anxiety**?

1 Yes

0 No

2) A consulting mental health clinician:

q192c Do any providers in your ACO use a mental health clinician (not co-located) consulting primary care clinicians to integrate primary care and treatment for **depression and/or anxiety**?

1 Yes

0 No

3) A patient registry to track mental health symptoms:

q192d Do any providers in your ACO use a patient registries to track mental health symptoms to integrate primary care and treatment for **depression and/or anxiety**?

1 Yes

0 No

The first question about use of care managers for mental health needs was the question that included an error on the web version (the text read “physical health needs” versus “mental health needs”). We considered all those who responded to this question on the web version as missing, and then combined the responses to questions q192a and q192b, e.g. use of a care manager for non-medical needs OR mental health needs, to derive an analytic variable to indicate whether organizations reported useof care manager, either for mental health or non-medical needs. We considered “full” implementers of collaborative care to be those ACOs who reported using all three of these strategies : 1) use of a care manager (either or mental health or non-medical needs), 2) use of a consulting mental health clinician, or 3) use of a patient registry to track mental health symptoms.

**Explanatory Variables : ACO Contract and Organizational Characteristics**

All explanatory/independent variables included in analyses were operationalized from survey questions specific to the contract and organizational characteristics of ACOs.

**Payer**

The first ACO contract variable we included was to identify the payer with whom respondents had a contract. We created three indicator variables to identify ACOs who had contracts with Medicare, those who had contracts with commercial payers, and those who had an ACO arrangement with Medicaid.

For ACOs with commercial contracts, we both asked whether organizations had an ACO contract or if they had a contract in which they were responsible for the total cost of care and quality performance for an attributed patient population as an additional sensitivity measure:

qs1a. Do you have an ACO/ACO-like contract with a commercial payer or private insurance plan at this time?

Please note: We consider all contracts that hold organizations responsible for the total cost of care and quality performance for a defined patient population to be ACO contracts.

1 Yes

2 No

qs1d. Are you responsible for the total cost of care for your patient population (e.g., through withholds, bonuses, shared savings, capitation) for any of your contracts with commercial insurance plans?

1 Yes

0 No

qs1e. Under your commercial contract where you are responsible for the total cost of care, are you responsible for quality performance?

1 Yes

0 No

We asked respondents whether they were currently participating in any Medicare program and considered all those who were participating in a Medicare program to have a Medicare contract (e.g., did not differentiate between types of Medicare contracts). In addition to asking respondents which program they participated in, we also had a drop-down list from which respondents could select their ACO’s name, as provided in Medicare’s participant list.

s120 Are you currently participating in any Medicare ACO program?

1 Yes, MSSP Track 1

2 Yes, MSSP Track 2

3 Yes, MSSP Track 1+

4 Yes, MSSP Track 3

5 Yes, Next Generation

6 No

We asked respondents to identify whether they participated in any Medicaid ACO arrangement. We identified respondents as having a contract with Medicaid if they reporting participating in any of Medicaid arrangements included in this question.

s124 Do you have an ACO/ACO-like contract for your **Medicaid patients** at this time? If so, in the table below please provide the information for each of your Medicaid ACO/ACO-like arrangements.

Please note: We consider all contracts that hold organizations responsible for the total cost of care and quality performance in a defined patient population to be ACO contracts.

**Previous Experience in Risk-Bearing Contracts**

The second ACO contract characteristic we included was previous experience in risk-bearing contracts. We asked the following questions in a grid. We considered respondents as having any experience if all or some of their participating providers identified previous participation in any of the following programs.

q5 Has your ACO or any of its participating providers previously joined any bundled payment or episode based payments, Medicare Advantage, Capitated commercial contracts, or other risk-bearing contracts? *(Participating providers are those to whom patients are attributed in the ACO.)*

1 Yes

2 No

**Financial Risk**

The third ACO contract characteristic we included was a measure indicating whether or not the ACO took on downside risk in their contract. Because responsibility for downside risk is payer and contract specific, we asked ACOs to report whether they were responsible for shared losses in their commercial or Medicaid contracts:

q7. What is the risk arrangement for your LARGEST **commercial** ACO contract in the current performance year?

1 We are responsible for shared losses (downside risk)

2 We have only shared savings or bonuses (no downside risk)

qm7. Which of the following risk arrangements best represents your LARGEST **Medicaid** ACO contract in the current performance year?

1 We are responsible for shared losses (downside risk)

2 We have only shared savings or bonuses (no downside risk)

We also considered ACOs participating in Medicare Shared Savings Program Track 1+, Track 2, Track 3 or Next Gen as taking on downside risk. We then created an aggregate measure that identified respondents who took on downside risk in any contract using the responses to q7 and qm7 as well as qs120 (participation in Medicare program).

**ACO Contract Characteristics specific to Mental Health**

Our final ACO contract characteristics included in our model were those characteristics that could have mental-health specific variation : the performance measures included in contract, the types of services included in the total cost of care calculation, or whether the respondent carved or contracted out mental health services entirely from its contract.

Behavioral health specific performance measures: All Medicare ACO contracts are required to report 33 measures, of which 2 are related to mental health care quality (ACO 18- Depression Screening and Follow-up, and ACO 40 – Depression Remission and Response). However, this requisite set of measures does not exist for commercial or Medicaid contracts, leaving payers and providers more discretion to choose/negotiate the performance measures included in contract. Therefore, we asked respondents who reported having either commercial or Medicaid ACO contracts to report whether their contracts included mental health quality measures. We then created an aggregate measure where 1 indicated all organizations who had a Medicaid or commercial contract and included mental health quality measures in this contract and where 0 indicated all organizations who had a Medicaid or commercial contract and did not include mental health quality measures in their contract, or only had a Medicare contract.

q168 **We are interested in behavioral health quality measures in your ACO’s commercial contracts.**

Are mental health quality measures included in your LARGEST **commercial** ACO contract?

1 Yes

0 No

q172 **We are interested in behavioral health quality measures in your ACO’s Medicaid contracts.**

Are behavioral health quality measures included in your LARGEST **Medicaid** ACO contract?

1 Yes

0 No

Carve-outs: Though Medicare Shared Savings Program participants are not allowed to contract out mental health services, commercial and Medicaid ACO contracts also have the discretion to contract out services to managed behavioral health care organizations – a phenomenon known as “carving out.” We asked ACOs who had contracts with commercial or Medicaid to report whether they carved out mental health services from their contracts. As with our quality variable, we then derived an aggregate indicator variable that identified all organizations with either Medicaid or Commercial contracts who reported carving out mental health services from that contract. Organizations who only had Medicare contracts were considered to not carve out mental health services. We treated Don’t Know responses as equivalent to “No”.

q156 In some cases, **mental health or addiction treatment** is contracted out or “carved out” to organizations like Beacon Health Strategies, New Point, or Magellan. Does this describe the arrangement for your LARGEST **commercial** ACO contract?

1 Yes

2 No

3 Don’t Know

q158 In some cases, **mental health or addiction treatment** is contracted out or “carved out” to organizations like Beacon Health Strategies, New Point, or Magellan. Does this describe the arrangement for your LARGEST **Medicaid** ACO contract?

1 Yes

2 No

3 Don’t Know

Total Cost of Care Calculation: Medicare Shared Savings Program participants are required to include mental health services when calculating the total cost of care for their attributed beneficiaries, but do not include prescription drug costs. We asked whether organizations with commercial or Medicaid contracts included mental health services in their total cost of care calculation. We then derived an aggregate variable that identified all organizations who had a commercial or Medicaid contract and included mental health services in the total cost of care calculation. We considered organizations who only had Medicare contracts as not including mental health services in the total cost of care. We treated Don’t Know as equivalent to No.

q6o. For your LARGEST **commercial** ACO contract, are mental health services included in the total cost of care calculation to determine any shared savings?

1 Yes

0 No

888 Don't know

qm6q.For your LARGEST **Medicaid** ACO contract, are mental health services included in the total cost of care calculation to determine any shared savings?

1 Yes

0 No

888 Don't know

**Structural Characteristics**

**Leadership Structure**

Physician led organizations are associated with improved financial performance, and smaller/ more nimble management/structuring of their ACO. We asked ACOs to report the leadership structure of their ACO and created an indicator variable that identified physician-led organizations (phys_led_nohosp = 1) compared to organizations that were not physician-led (phys_led_nohosp = 0). We considered organizations who reported being jointly led as not being physician led. Because many respondents may report being physician led if physicians are included on their boards, we further restricted this variable by requiring physician-led organizations to report NOT including hospitals in their ACO network (1=Physician-led, no hospital; 0 = Not physician led, or physician-led with hospital) .

q38. Which of the following best describes the leadership structure of your ACO?

1 Physician-led

2 Hospital-led

3 Jointly led by physicians and hospital

4 Coalition-led

5 State, region, or county-led

6 Other [q38_spec] – Please specify: [TEXT BOX]

**Size**

We asked organizations to report how many full-time equivalent primary care and

specialty clinicians were participating in the respondents largest contract. If the respondent had a

Medicare Shared Savings Program contract, we replaced the self-reported number with the

number for FTE primary care clinicians and specialty clinicians reported in CMS 2017 MSSP

public use files. We then summed responses to these questions to develop a total number of

FTEs and then created a four-part categorical variable representing quartiles of total clinician

FTEs.

q48. Approximately how many full–time equivalent (FTE) **primary care clinicians** are participating in your largest ACO contract?

*(For the purposes of this survey, participating primary care clinicians are those to whom patients can be attributed in the ACO, including primary care physicians [internists, family medicine, pediatrics, geriatricians], physician assistants, and nurse practitioners.)*

1 __________ FTE Primary Care Clinicians [RANGE: 0-9000]

q50. Approximately, how many full-time equivalent (FTE) **specialty clinicians** are participating in your largest ACO contract?

*(For the purposes of this survey, participating specialty clinicians includes physician assistants and nurse practitioners.)*

__________ FTE Specialty Clinicians [RANGE: 0-9000]

**Section 2: Statistical Technical Appendix**

**Measuring collaborative care**

Using responses from the 405 organizations who answered questions on collaborative care in the Wave 4 NSACO, we measured the proportion of organizations who reported using each collaborative care strategies to integrate mental health and primary care for patients with depression or anxiety: 1) care manager (either for mental health treatment coordination or non-medical needs), a consulting mental health clinician, and a patient registry to track mental health symptoms (q192a-q192d in the survey instrument). We then identified the proportion of organizations who reported using no strategies, those that only partially implemented collaborative care (reported use of 1-2 strategies), and those that reported full implementation (reported use of all 3 strategies). We plotted the intersection of these strategies using a Venn Diagram (Exhibit 1 in manuscript).

**Descriptive Statistics**

Using responses from the 405 organizations who answered questions on collaborative care in to the Wave 4 NSACO, we measured the distribution of each contract and organizational characteristic using responses to the Wave 4 NSACO. We compared the distribution of contract and organizational features by collaborative care implementation to observe the differences between ACOs with no reported implementation, those with just partial implementation, and those with full implementation (Table 1 in manuscript). We compared differences in proportions using F-tests and reported p-values in Exhibit 2. We considered a p-value less than 0.05 as statistically significant. We used multiply imputed data to account for organizations who had missing covariate information.

**Association between contract characteristics and collaborative care: repeated measures regression model**

**Method:** Each collaborative care strategy was measured as a 0,1 binary outcome where 1 indicated that the respondent was implementing that strategy and 0 indicated that the organization was not implementing that strategy. We combined these three outcomes to form one multivariate binary outcome (Y). We regressed this binary outcome on all contract and organizational characteristics using a logit model, considering each collaborative care strategy a repeated measure (j) within each ACO (i). We fit the model (Model 1.1) using generalized estimating equations (GEE)^[[1]](#footnote-1)^ and specified an unstructured correlation matrix with robust standard errors. Because the association between ACO characteristics and collaborative care implementation could change depending on the type of collaborative care strategy (CCStrategy), we created interaction terms between each ACO characteristic and collaborative care strategy variable ( a 3-level categorical variable that refers to type of collaborative care strategy where 1 refers to use of care managers, 2 refers to consulting mental health clinicians, and 3 refers to patient registries).

$$1.1 logit\left( Y_{ij} \right)=\beta_{0}+ \beta_{1}CCStrategy_{i}+ \beta_{2}Contract_{ij}+ \beta_{3}Leadership_{ij}+ \beta_{4}Size_{ij}+B_{5}Mode_{ij}+\beta_{6}CCStrateg{y*Contract}_{ij}+\beta_{7}CCStrategy*Leadership_{ij}+ \varepsilon_{i}$$

The covariate *Contract*  is a vector of indicator variables identifying contract characteristics, such as payers, sharing financial risk in any contract, inclusion of mental health services in total cost of care or in performance measures, and whether mental health services were carved out of ACO contract. Covariate *Leadership* is an indicator variable that identifies organizations that report being physician led versus not physician led. Covariate *Size* is a four part categorical variable referring to the quartile of size, as defined by total number of physician FTEs in an organization. Covariate *Mode* is a control variable included to adjust for the type of survey to which organizations responded (paper or web).

*Model fitting*

To improve the efficiency of the coefficients estimating the effect of ACO characteristics on collaborative care implementation (e.g. all the non-interaction terms), we tested the joint significance of the interaction term and the corresponding coefficient for each ACO characteristic.. We included all interaction terms where the Wald statistic passed the significance threshold of p<0.05 in test for joint significance in the final model. Below is the table the fitted coefficients and associated standard errors.

| **Table 1: Fitted Model** |  |
| --- | --- |
| VARIABLES | *coefficient* |
|  | *(standard error)* |
| **Collaborative Care Strategy Type** |  |
|  |  |
| Care Manager | 0.573* |
|  | (0.307) |
| Consulting MH Clinician | Reference Group |
| Patient Registry | -1.522*** |
|  | (0.310) |
| **Contract Features** |  |
| Has a Medicaid ACO Contract | 0.283 |
|  | (0.205) |
| Has a Medicare ACO Contract | -0.0593 |
|  | (0.220) |
| Has a Commercial ACO contract | -0.165 |
|  | (0.211) |
| Shares Financial Risk in Any Contract | 0.201 |
|  | (0.191) |
| Includes MH in Total Cost of Care | 0.194 |
|  | (0.195) |
| Include MH Services in Quality Measures | 0.699*** |
|  | (0.247) |
| Carves out MH Services from Contract | 0.268 |
|  | (0.199) |
| Has Previous Experience in risk-based Contracting | 0.0553 |
|  | (0.164) |
| Physician-Led | 0.0774 |
|  | (0.198) |
| Size in FTE (by Quartile) |  |
| Quartile 1 | -0.659** |
|  | (0.330) |
| Quartile 2 | -0.525* |
|  | (0.302) |
| Quartile3 | Reference Group |
| Quartile 4 | -0.0442 |
|  | (0.310) |
| Mode | -0.258 |
|  | (0.183) |
| Care Manager# 1.Quality | -0.340 |
|  | (0.338) |
| Patient Registry#1.Quality | -0.797*** |
|  | (0.308) |
| Care Manager # Size Quartile 1 | -0.332 |
|  | (0.392) |
| Care Manager # Size Quartile 2 | 0.0725 |
|  | (0.398) |
| Care Manager # Size Quartile 4 | 0.955** |
|  | (0.444) |
| Patient Registry # Size Quartile 1 | 0.498 |
|  | (0.389) |
| Patient Registry # Size Quartile 2 | 0.588 |
|  | (0.387) |
| Patient Registry # Size Quartile 4 | 0.359 |
|  | (0.417) |
| Constant | 0.327 |
|  | (0.343) |
|  |  |
| Observations | 1,207 |
| Number of acoid | 405 |
| Robust standard errors in parentheses |  |
| *** p<0.01, ** p<0.05, * p<0.1 |  |

**Rationale:** Leveraging our multivariate outcome and estimating the association between ACO contract characteristics on use of collaborative care using GEE makes this analysis more efficient. Other methods to understand the association between ACO characteristics and use of collaborative care could include estimating a restrictive logit model that would measure the association between ACO characteristics and full implementation of collaborative care, where 1 would indicate full implementation of collaborative care (reported use of all 3 collaborative care strategies), or to estimate a separate logit model for each collaborative care strategy (where 1 would indicate use of that particular strategy). However, these methods either exclude important information (in the case of the restrictive model), or ignore the fact that our outcomes of interest are correlated. The GEE model both accounts for the correlation between collaborative care outcomes, and provides more power to the analysis through increasing the total number of observations. ^[[2]](#footnote-2)^

*Correlation matrix*

The GEE procedure is robust to different specifications of the correlation matrix, but correctly specifying the correlation matrix does improve the efficiency of the GEE estimator. We specified an unstructured correlation matrix and computed the correlation matrix using the postestimation command estat with multiple models: 1) Simple model (just the type of collaborative care strategy variable included):

| c1 c2 c3

------+---------------------------------

r1 | 1

r2 | .2475286 1

r3 | .1313894 .2512503 1

The inclusion of additional covariates reduced the correlation between collaborative care outcomes:

| c1 c2 c3

------+---------------------------------

r1 | 1

r2 | .170616 1

r3 | .0848857 .2394694 1

Whereas including interaction terms back into the model inflated the correlations between outcomes again slightly:

| c1 c2 c3

------+---------------------------------

r1 | 1

r2 | .2002001 1

r3 | .0900509 .2361756 1

*Computing Predicted Probabilities*

After fitting our final model, we computed the predicted probability of each contract and organizational characteristic on the likelihood of implementing collaborative care. We held all variables at their observed values. Because all covariates were binary, we computed the predicted probability of implementing collaborative care with and without each characteristic and report the difference between these two point estimates (the average marginal effect) in Figure 2 in the manuscript using ggplot2 package in RStudio (version 1.2.1335).

**Section 3: Missing data procedures**

Because we were using survey data for this study, we did not have complete data for each respondent due to item non-response. To account for missing covariate information among all 405 respondents who responded to the collaborative care strategy questions in the 2017-2018 NSACO (q192a-d), we used a multiple imputation through chained equations (MICE) procedure to generate a “full” data set. Our imputation model was identical to our analytic model and we specified 10 imputed datasets.

As a comparison, we also created a complete case cohort of respondents, or respondents who had no missing observations for any of the variables included in our analytic model (either our outcome variable - collaborative care strategies – or any explanatory variables - payer, contract characteristics, leadership or size). This reduced our total number of observations by approximately 13%, from 405 observations to 354 observations. Using the complete case cohort did not change the prevalence of our primary outcome (use of collaborative care), but did change the effect size and direction of effect for some covariates included in our model:

**Figure1: Comparison of Completed Case and Multiply Imputed Data on Analysis**

**
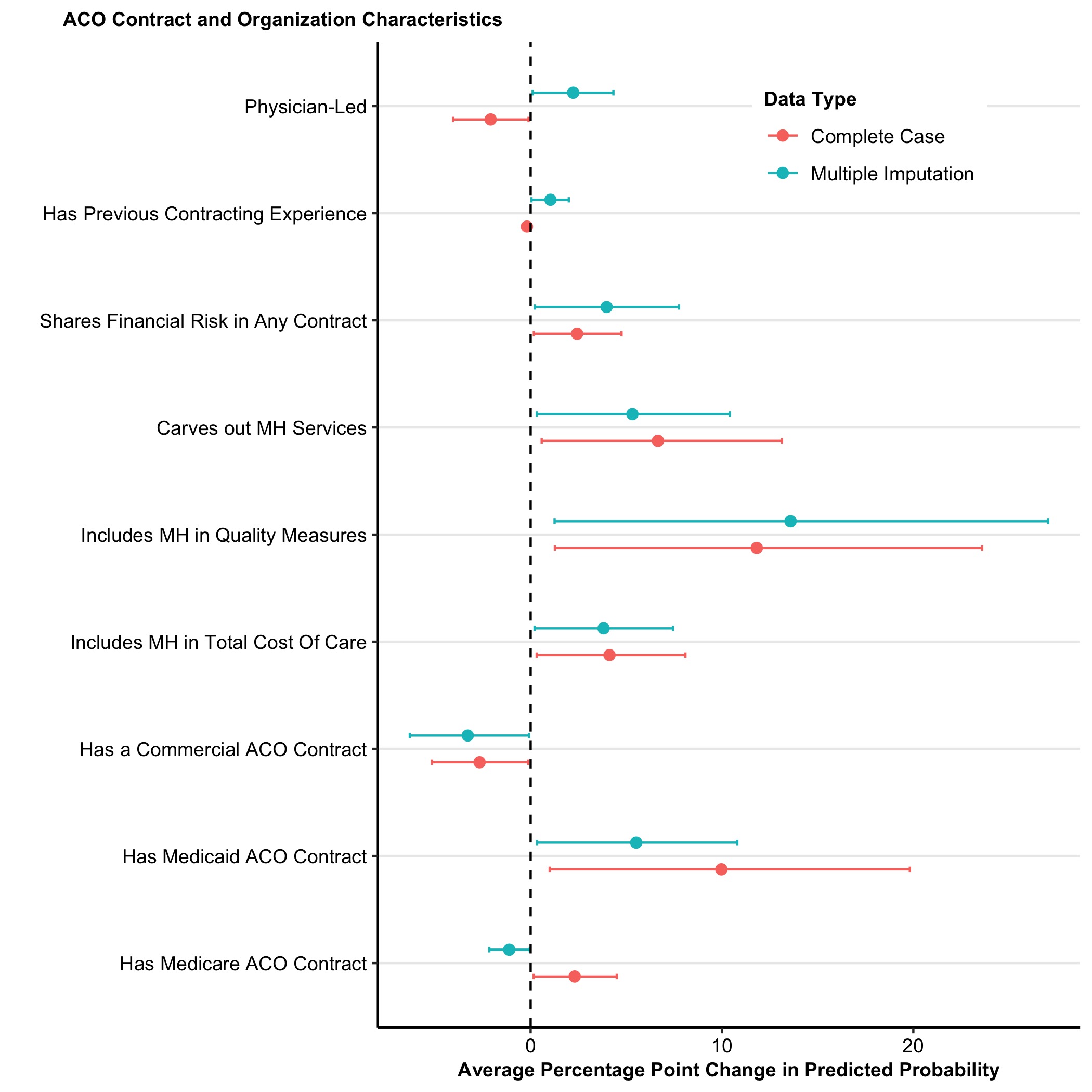
**

**Section 4: Sensitivity Analyses for Care Management Outcome**

We also conducted a sensitivity analysis to examine where excluding the question that had the wording error (q192a) affected the results from our analysis. Excluding q192a from our definition of care manager reduced the overall proportion of care managers by 3 percent (from 71% to 68%), but did not change the association between ACO characteristics and use of collaborative care (see Figure 2). As an additional test of robustness, we also examined our model to see if our results were robust to the exclusion of the care-manager outcome entirely. Table 2 shows the comparison in model results from the original model, model excluding q192a, and the model excluding all care manager questions (both q192a and b). Our estimates were similar across all models.

**Figure2: Sensitivity analysis excluding q192a from care manager definition**

**
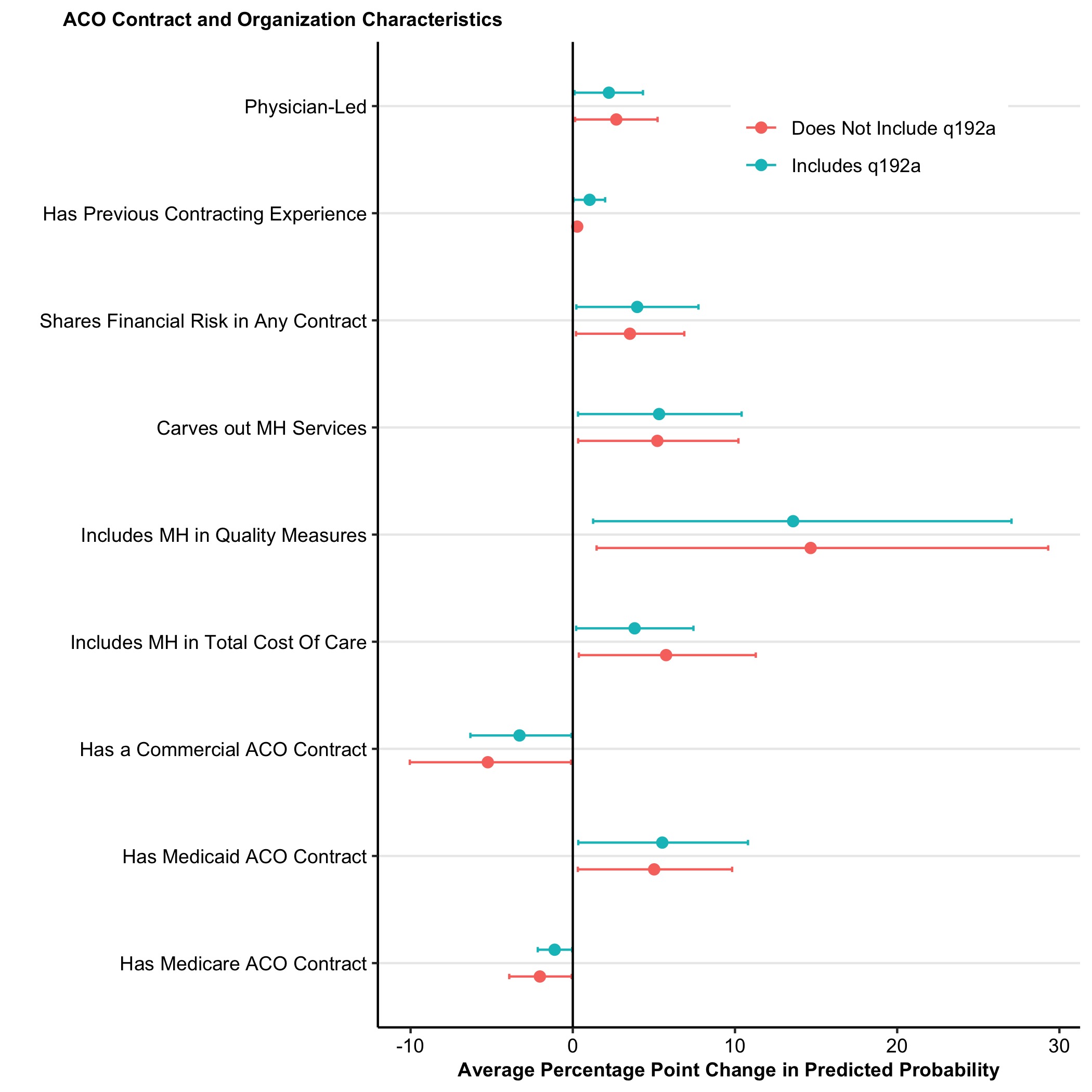
**

| **Table 2: Adjusted Associations between ACO characteristics and collaborative care implementation** | | | | | | | | | |
| --- | --- | --- | --- | --- | --- | --- | --- | --- | --- |
|  | **Model A:**  Excludes q192a and q192b | | | **Model B:**  Excludes q192a | | | **Original Model** | | |
| **ACO Characteristics** | **Mean PP Change** | LCI | UCI | **Mean PP Change** | LCI | UCI | **Mean PP Change** | LCI | UCI |
| **Payer** |  |  |  |  |  |  |  |  |  |
| Has a Medicare Contract | **-0.162** | -0.017 | -0.311 | **-0.150** | 0.001 | -0.277 | **-0.144** | 0.001 | -0.265 |
| Has a Medicaid Contract | **0.002** | 0.000 | 0.004 | **0.002** | 0.000 | 0.004 | **0.002** | 0.000 | 0.003 |
| Has a Commercial Contract | **-0.065** | -0.010 | -0.129 | **-0.066** | -0.003 | -0.125 | **-0.070** | -0.002 | -0.131 |
| **Contract Characteristics** |  |  |  |  |  |  |  |  |  |
| Includes mental health services in total cost of care calculation | **0.114** | 0.025 | 0.237 | **0.119** | 0.015 | 0.236 | **0.122** | 0.015 | 0.240 |
| Includes any mental health quality measures in contract measure set | **0.125** | 0.028 | 0.263 | **0.132** | 0.018 | 0.261 | **0.133** | 0.017 | 0.264 |
| Carves out mental health services from contract | **0.065** | 0.013 | 0.134 | **0.067** | 0.007 | 0.131 | **0.068** | 0.006 | 0.132 |
| Shares financial risk in any contract^^ | **0.026** | 0.005 | 0.054 | **0.026** | 0.002 | 0.049 | **0.021** | 0.002 | 0.041 |
| Previous experience in risk-based contracts | **0.029** | 0.005 | 0.053 | **0.028** | 0.002 | 0.047 | **0.026** | 0.002 | 0.044 |
| **Organizational Characteristics** |  |  |  |  |  |  |  |  |  |
| Physician-led ACO^^ | **0.006** | 0.001 | 0.012 | **0.002** | 0.000 | 0.005 | **0.004** | 0.000 | 0.008 |
| **Size (# Clinician FTEs)** |  |  |  |  |  |  |  |  |  |
| Very Small | **-0.152** | -0.151 | -0.278 | **-0.135** | -0.148 | -0.238 | **-0.132** | -0.145 | -0.232 |
| Small | **-0.087** | -0.089 | -0.156 | **-0.075** | -0.085 | -0.130 | **-0.077** | -0.088 | -0.134 |
| Medium (average) | Reference group | |  | Reference group | |  | Reference group | |  |
| Large | -0.005 | -0.005 | -0.009 | -0.006 | -0.007 | -0.009 | -0.004 | -0.005 | -0.007 |
| **Note:** PP refers to percentage point change, LCI is lower confidence interval, UCI is upper confidence interval. Each model was a logistic regression fit with generalized estimating equations with an unstructured correlation matrix and robust standard errors. The model specification included all covariates in this table, the type of survey taken (web/paper) and interaction terms between the type of strategy and each ACO characteristic. | | | | | | | | | |

| **Table 3: Characteristics of 2017 Medicare Shared Savings Program ACOs by NSACO Respondent Status** | | | | | | |
| --- | --- | --- | --- | --- | --- | --- |
|  | **Non-Respondents** |  | **Respondents** | |  | **P-Value** |
| **N** | (165 ACOs) |  | (306 ACOs) | |  |  |
| **Census Region** |  |  | |  |  | 0.59 |
| Northeast | 29 | 18% | | 61 | 20% |  |
| Midwest | 32 | 19% | | 68 | 22% |  |
| South | 70 | 42% | | 113 | 37% |  |
| West | 16 | 10% | | 36 | 12% |  |
| NE & South | 6 | 4% | | 8 | 3% |  |
| South & MW | 7 | 4% | | 11 | 4% |  |
| West & NE | 1 | 1% | | 0 | 0% |  |
| West & MW | 0 | 0% | | 3 | 1% |  |
| South & West | 1 | 1% | | 3 | 1% |  |
| S, MW, NE | 1 | 1% | | 1 | 1% |  |
| S, W, MW | 2 | 1% | | 0 | 0% |  |
| S, MW, W, NE | 0 | 0% | | 1 | 1% |  |
|  |  |  | |  |  |  |
| **Demographics** |  |  | |  |  |  |
| No. Attributed lives | 18,429 | 1521 | | 19,391 | 1080 | 0.6 |
| Percent Female | 57 | (57.1-57.7) | | 57 | (56.9-57.3) | 0.28 |
| **Race** |  |  | |  |  |  |
| Percent White | 78 | (75.5-81.4) | | 87 | (85.6-87.9) | <0.0001 |
| Percent Black | 12 | (10.4-14.6) | | 8 | (6.7-8.6) | <0.0001 |
| Percent Asian | 3 | (1.6-4.9) | | 1 | (0.9-1.2) | 0.0004 |
| Percent Hispanic | 2.3 | (1.7-2.9) | | 1.6 | (1.2-1.9) | 0.03 |
| **Age Distribution** |  |  | |  |  |  |
| Percent under 65 | 17 | (15.7-18.1) | | 16 | (15.4-17.0) | 0.28 |
| Percent over 85 | 12 | (11.4-12.5) | | 12 | (11.6-12.2) | 0.94 |
| **Providers** |  |  | |  |  |  |
| No. CAHs | 1.03 | (0.2-1.9) | | 1.4 | (0.99-1.8) | 0.38 |
| No. FQHCs | 5.8 | (1.9-9.7) | | 4.6 | (2.7-6.3) | 0.53 |
| No. PCPs | 232 | (184-279) | | 240 | (210-271) | 0.76 |
| No. Specialists | 350 | (261-438) | | 413 | (347-481) | 0.26 |
| **Utilization** |  |  | |  |  |  |
| Inpatient Admissions | 333 | (322-344) | | 321 | (312-329) | 0.08 |
| Total E&M Visits | 10691 | (10419-10963) | | 10325 | (10131-10520) | 0.03 |
| **Source:** 2017 Medicare Shared Savings Program Public Use File | | | | | | |
| **Abbreviations:** | CAH | Critical Access. Hospital | | | |  |
|  | FQHC | Federally Qualified Health Center | | | | |
|  | PCP | Primary Care Provider | | | |  |
|  | E&M | Evaluation & Management | | | |  |

| **Table 4: Characteristics of NSACO respondents by survey mode (web vs. paper)** | | | | |
| --- | --- | --- | --- | --- |
|  | **Whole Sample** | **Survey Mode** | |  |
|  |  | **Web** | **Paper** | **P-Value** |
| **Contract Characteristics** | (405 ACOs, 100%) | (323 ACOs, 80 %) | (82 ACOs, 20%) |  |
| **Payer** N (%) |  |  |  |  |
| *(Most ACOs have contracts with 2 or more payers)* |  |  |  |  |
| Has a Medicare Contract | 338 (83%) | 274 (85%) | 64(78%) | 0.14 |
| Has a Commercial Contract | 295 (73%) | 233 (72%) | 62 (75%) | 0.53 |
| Has a Medicaid Contract | 96 (24%) | 77 (23%) | 19(20%) | 0.89 |
| **Financial Characteristics** N (%) |  |  |  |  |
| Shares financial risk in any contract | 149 (37%) | 111 (35%) | 38 (46%) | 0.05 |
| Previous experience in risk-based contracts | 257 (64%) | 204 (54%) | 53 (66%) | 0.68 |
| **Mental Health Contract Characteristics** N (%) |  |  |  |  |
| *(included in non-Medicare contracts)* |  |  |  |  |
| Includes mental health services in total cost of care calculation | 163 (43%) | 136 (29%) | 27 (44%) | 0.10 |
| Includes mental health in quality performance measures | 134 (34%) | 113 (35%) | 21 (26%) | 0.13 |
| Carves out mental health services from contract | 84 (21%) | 66 (21%) | 18 (22%) | 0.73 |
| **Organizational Characteristics** |  |  |  |  |
| **Leadership** N (%) |  |  |  |  |
| Physician-led ACO | 143 (38%) | 118 (39%) | 25 (31%) | 0.19 |
| **Partnerships** N (%) |  |  |  |  |
| Includes specialty behavioral health provider in ACO network | 54 (13%) | 40 (13%) | 14 (17%) | 0.29 |
| Includes Federally Qualified Health Center (FQHC) in ACO network | 104 (26%) | 87 (27%) | 17 (21%) | 0.22 |
| Includes academic medical center in ACO network | 72 (18%) | 49 (15%) | 23 (28%) | 0.01 |
| Includes public hospital in ACO network | 45 (11%) | 36 (11%) | 9 (11%) | 0.93 |
| **Size** mean (95% CI) |  |  |  |  |
| Number of clinicians in ACO network | 797 (679-915) | 803 (664-942) | 753 (553-953) | 0.74 |
| **Source:** 2017-2018 NSACO | | | | |

| **Table 5: Model Estimates by Survey Mode** | |  |  |
| --- | --- | --- | --- |
|  | Full Sample | Respondents who answered using web survey | Respondents who answered using paper survey |
| VARIABLES | *coefficient* |  |  |
|  | *(standard error)* |  |  |
| **Collaborative Care Strategy Type** |  |  |  |
|  |  |  |  |
| Care Manager | 0.573* | 0.612* | 0.859* |
|  | (0.307) | (0.371) | (0.452) |
| Consulting MH Clinician | Reference Group |  |  |
| Patient Registry | -1.522*** | -1.134*** | -3.776*** |
|  | (0.310) | (0.326) | (1.067) |
| **Contract Features** |  |  |  |
| Has a Medicaid ACO Contract | 0.283 | 0.269 | 0.443 |
|  | (0.205) | (0.227) | (0.612) |
| Has a Medicare ACO Contract | -0.0593 | -0.160 | 0.458 |
|  | (0.220) | (0.273) | (0.449) |
| Has a Commercial ACO contract | -0.165 | -0.0743 | -0.728 |
|  | (0.211) | (0.250) | (0.498) |
| Shares Financial Risk in Any Contract | 0.201 | 0.190 | 0.0136 |
|  | (0.191) | (0.223) | (0.449) |
| Includes MH in Total Cost of Care | 0.194 | 0.160 | 0.719 |
|  | (0.195) | (0.226) | (0.520) |
| Include MH Services in Quality Measures | 0.699*** | 0.744*** | 0.711 |
|  | (0.247) | (0.275) | (0.746) |
| Carves out MH Services from Contract | 0.268 | 0.157 | 0.793 |
|  | (0.199) | (0.234) | (0.504) |
| Has Previous Experience in risk-based Contracting | 0.0553 | -0.0159 | 0.589 |
|  | (0.164) | (0.182) | (0.475) |
| Physician-Led | 0.0774 | 0.153 | -0.284 |
|  | (0.198) | (0.233) | (0.431) |
| Size in FTE (by Quartile) |  |  |  |
| Quartile 1 | -0.659** | -0.738** | -0.158 |
|  | (0.330) | (0.368) | (0.831) |
| Quartile 2 | -0.525* | -0.309 | -0.990 |
|  | (0.302) | (0.352) | (0.713) |
| Quartile3 | Reference Group |  |  |
| Quartile 4 | -0.0442 | -0.100 | 0.0437 |
|  | (0.310) | (0.348) | (0.772) |
| Mode | -0.258 |  |  |
|  | (0.183) |  |  |
| Care Manager# 1.Quality | -0.340 | -0.271 | -0.791 |
|  | (0.338) | (0.392) | (0.724) |
| Patient Registry#1.Quality | -0.797*** | -1.038*** | -0.117 |
|  | (0.308) | (0.331) | (0.933) |
| Care Manager # Size Quartile 1 | -0.332 | -0.411 | -0.164 |
|  | (0.392) | (0.457) | (0.747) |
| Care Manager # Size Quartile 2 | 0.0725 | -0.383 | 0.680 |
|  | (0.398) | (0.477) | (0.675) |
| Care Manager # Size Quartile 4 | 0.955** | 0.816 | 1.085 |
|  | (0.444) | (0.521) | (0.825) |
| Patient Registry # Size Quartile 1 | 0.498 | 0.367 | Omitted (0) |
|  | (0.389) | (0.406) |  |
| Patient Registry # Size Quartile 2 | 0.588 | -0.0726 | 3.383*** |
|  | (0.387) | (0.435) | (1.147) |
| Patient Registry # Size Quartile 4 | 0.359 | 0.0785 | 2.172 |
|  | (0.417) | (0.433) | (1.364) |
| Constant | 0.327 | 0.404 | -0.413 |
|  | (0.343) | (0.404) | (0.812) |
|  |  |  |  |
| Observations | 1,207 | 964 | 229 |
| Number of acoid | 405 | 323 | 82 |
| Robust standard errors in parentheses |  |  |  |
| *** p<0.01, ** p<0.05, * p<0.1 |  |  |  |

1. To learn more about GEE, see Liang et. al. 2011. To learn more about the efficiency gains in estimating effects for a series of regression equations, see discussion on seemingly unrelated regression equations (SURE) by Zellner et. al. 2012. [↑](#footnote-ref-1)
2. The efficiency gain through leveraging a multivariate outcome is explained in greater detail in discussion on seemingly unrelated regression equations (SURE) by Zellner et. al. 2012. [↑](#footnote-ref-2)
